# Women experience a better long-term immune recovery and a better survival on HAART in Lao People’s Democratic Republic

**DOI:** 10.1186/1471-2334-13-27

**Published:** 2013-01-22

**Authors:** Mathieu Bastard, Khamphang Soulinphumy, Prasith Phimmasone, Ahmed Hassani Saadani, Laura Ciaffi, Arlette Communier, Chansy Phimphachanh, René Ecochard, Jean-François Etard

**Affiliations:** 1 Epicentre, Paris, France; 2 Ministry of Health/HIV Unit, Savannakhet Hospital, Savannakhet, Lao PDR; 3 Mahosot Hospital, Vientiane, Lao PDR; 4 Institut de la Francophonie pour la Médecine Tropicale, Vientiane, Lao PDR; 5 Médecins Sans Frontières, Geneva, Switzerland; 6 Ministry of Health – Centre for HIV, AIDS and STIs, Vientiane, Lao PDR; 7 Hospices Civils de Lyon, Service de Biostatistique, Lyon, F-69003, France; 8 Université Lyon I, Villeurbanne, F-69100, France; 9 CNRS, UMR 5558, Laboratoire de Biométrie et Biologie Evolutive, Equipe Biostatistique Santé, Pierre-Bénite, F-69310, France; 10 UMI 233 TransVIHMI, Institut de Recherche pour le Développement, Université Montpellier 1, Montpellier, France

## Abstract

**Background:**

In April 2003, Médecins Sans Frontières launched an HIV/AIDS programme to provide free HAART to HIV-infected patients in Laos. Although HIV prevalence is estimated as low in this country, it has been increasing in the last years. This work reports the first results of an observational cohort study and it aims to identify the principal determinants of the CD4 cells evolution and to assess mortality among patients on HAART.

**Methods:**

We performed a retrospective database analysis on patients initiated on HAART between 2003 and 2009 (CD4<200cells/μL or WHO stage 4). We excluded from the analysis patients who were less than 16 years old and pregnant women. To explore the determinants of the CD4 reconstitution, a linear mixed model was adjusted. To identify typical trajectories of the CD4 cells, a latent trajectory analysis was carried out. Finally, a Cox proportional-hazards model was used to reveal predictors of mortality on HAART including appointment delay greater than 1 day.

**Results:**

A total of 1365 patients entered the programme and 913 (66.9%) received an HAART with a median CD4 of 49 cells/μL [IQR 15–148]. High baseline CD4 cell count and female gender were associated with a higher CD4 level over time. In addition, this gender difference increased over time. Two typical latent CD4 trajectories were revealed showing that 31% of women against 22% of men followed a high CD4 trajectory. In the long-term, women were more likely to attend appointments without delay. Mortality reached 6.2% (95% CI 4.8-8.0%) at 4 months and 9.1% (95% CI 7.3-11.3%) at 1 year. Female gender (HR=0.17, 95% CI 0.07-0.44) and high CD4 trajectory (HR=0.19, 95% CI 0.08-0.47) were independently associated with a lower death rate.

**Conclusions:**

Patients who initiated HAART were severely immunocompromised yielding to a high early mortality. In the long-term on HAART, women achieved a better CD4 cells reconstitution than men and were less likely to die. This study highlights important differences between men and women regarding response to HAART and medical care, and questions men’s compliance to treatment.

## Background

Highly active antiretroviral therapy (HAART) has clearly reduced morbidity and mortality of HIV-infected patients both in industrialized and developing countries [1-3]. The majority of HIV-infected people live in resource-poor settings where programmes have been launched to provide free HAART. The efficacy and the feasibility of such programmes have been largely proved [4-6]. In 2009, antiretroviral therapy coverage was estimated at 37% in Sub-Saharan Africa and 31% in East, South and Southeast Asia [7].

In Asia, the first case of AIDS was reported in 1984. The actual HIV prevalence is low but several countries show growing HIV epidemics [7,8]. In Lao People’s Democratic Republic (Lao PDR), the first HIV infected patient was detected in 1990 but the HIV epidemiology is still not very well documented. The overall prevalence seems to have increased over the past years and is estimated at 0.2% [0.2; 0.4%] in 2009 but this figure could be underestimated given the very few data available [7]. The epidemic is largely driven by heterosexual transmission and cross-border migration is recognized as a key factor of the dynamic of the epidemic. Several studies and reports show a higher prevalence among men who have sex with men (5.6%), female migrants (0.8%) and service women (0.4%) [9-12]. In order to prevent HIV infection, a 100% Condom Use Programme and the HIV/AIDS/STIs Plan for Lao PDR with twelve priority strategies have been developed [13,14].

In July 2001, Médecins Sans Frontières (MSF) opened a HIV/AIDS project in Savannakhet Provincial Hospital in Lao PDR to provide prophylaxis and treatment for opportunistic infections for HIV-infected patients. In April 2003, MSF started a programme of free antiretroviral distribution in Savannakhet Hospital which became at that time the only place in the country to provide HAART to HIV/AIDS-infected patients.

Several studies on cohorts of HIV-infected patients receiving HAART in Thailand and Cambodia have been published but to date, there is no equivalent publication on the outcomes of patients on-HAART in Lao PDR [15-20]. To fill this gap, we conducted a retrospective analysis on a database made available to us by the Ministry of health/Center for HIV and STI and MSF in order to report the first results on patients receiving HAART at Savannakhet Provincial Hospital, located on the Thai-Lao border, between April 2003 and June 2009. This work aims at assessing immune recovery and survival of patients on HAART.

## Methods

### Patients

Patients included in this retrospective analysis entered the MoH-MSF programme of free HAART distribution between April 2003 and June 2009. We excluded from analysis patients who were less than 16 years old and pregnant women. Patients started on HAART if they met one of the following criteria: (1) CD4 cell count < 200 cells/μL irrespective of WHO clinical stage; (2) WHO clinical stage 4 irrespective of CD4 cell count. Date of HAART initiation for eligible patients ranged between September 2003 and June 2009.

Main initial regimens were triple-drug combinations made of two nucleoside reverse transcriptase inhibitor + one non-nucleoside reverse transcriptase inhibitor.

HIV testing, HAART, treatment of opportunistic infections (including tuberculosis), laboratory monitoring and transportations of patients were free of charge for eligible patients.

### Follow-up of the patients

After a first visit at HAART initiation, patients were followed-up at week 2 and 4, and then, once a month at Savannakhet Provincial Hospital. After 6 months on HAART and if there were no complaints from the patients, the follow-up took place every 3 months. The CD4 cell count was monitored every 6 months, but no measurement of viral load was available. A paper-based system was used by the medical team to allow the clinical follow-up of the patients. Some key data on patients and follow-up were collected to produce activity reports using FUCHIA 1.6.1 software (Epicentre, Paris).

In October 2006, MSF opened a second treatment centre in capital Vientiane allowing the transfer of pre-HAART and on-HAART patients who originally lived in or around Vientiane. No information on patients transferred to Vientiane could be retrieved so they were censored at date of transfer. We considered a patient as lost to follow-up if their last visit preceded the closure date of the database by 6 months and if he was not dead and had not been transferred to Vientiane.

### Data analysis

We performed a retrospective analysis on anonymized data, captured with Fuchia, made available to us by MoH-MSF. An internal unique key was generated by the system to link the patients’ visits so that it was impossible to identify a patient. The database was locked on 30^th^ June 2009. The CD4 cell counts not available at HAART initiation (D_0_) were retrieved from 15 days before to 15 days after D_0_, and in a median time of 34 days before D_0_ for those with still missing CD4 cell count.

Baseline characteristics of men and women were compared using Fisher’s exact test for categorical variables and Wilcoxon test for continuous variables.

We estimated the median progression of CD4 cells on HAART. We fitted a random-intercept linear mixed model for repeated measures using all available CD4 measurements at each time point to explore the effect of several characteristics of patients at HAART initiation: age, gender, Body mass index (BMI), WHO clinical stage, CD4 cell count and haemoglobin level. We also fitted a latent trajectory model, both for men and women, with a latent variable characterizing different patterns of CD4 cells over time [21]. This method aims at identifying typical latent trajectories of CD4 cells based on individual CD4 cells measures over time, as well as the prevalence of each latent trajectory. To model these trajectories, a polynomial function of time on HAART was considered. Then, we estimated, for each patient, a membership probability to each trajectory, and we assigned a unique trajectory to a patient by taking the maximum membership probability. Each patient has their own immune recovery over time, so it is important to underline that one typical trajectory does not describe entirely a given patient’s CD4 evolution. This kind of analysis may be affected by the number of CD4 measurements over time per patient. To validate this approach, we have analyzed the membership probabilities according to the number of CD4 measurements for each patient.

We also analyzed the punctuality of scheduled appointments, defined as attending the visit the exact scheduled day (or before), over time on HAART using mixed-effect logistic regression.

The participation time on HAART was calculated as the time between D_0_ and the date of last visit, the date of transfer or the date of death. We described the mortality on HAART using Kaplan-Meier curves and log-rank tests. A Cox proportional-hazards model was adjusted to explore the link between typical latent CD4 trajectories and mortality, thus baseline CD4 count was not included because its effect was captured by the latent trajectories. Other possible risk factors at HAART initiation such as age, gender, Body mass index (BMI), WHO clinical stage and haemoglobin level were considered in the analysis. Punctuality of scheduled appointments was also investigated. Proportional hazards (PH) assumption was checked by testing the Schoenfeld residuals. Because gender appeared to violate the PH assumption, time was split at 9 months and the effect of gender was thus estimated independently before and after 9 months on HAART.

Statistical analyses were performed with Stata 10.1 (Stata Corporation, College Station, Texas, USA) software using the generalized linear latent and mixed model (GLLAMM) framework [22].

### Ethical clearance

This research followed the principles of the Helsinki Declaration, 2004 amendment and analysis plan was approved by the Ministry of Health – Centre for HIV, AIDS and STIs (MoH/CHAS) of Lao PDR which granted access to the anonymized data. This retrospective data analysis was performed on routinely collected data which were fully anonymized and involved no risk for participants. Verbal informed consent was obtained from patients during the first visit, and therefore, their first inclusion into the database.

## Results

A total of 1365 patients were registered in the database. Among them, 3 were excluded because there were no data recorded for them and 134 were excluded because they were less than 16 years old. Before receiving HAART, 127 patients died, 30 were transferred to Vientiane and 71 were lost to follow-up. Among the 1000 patients alive and followed, 913 (91.3%) were started on HAART and included in the analyses where as 87 were not initiated on ART because they were not eligible at the closing date of the database.

Patients’ characteristics at HAART initiation are presented in Table 1. Median age was 32 years [interquartile range (IQR) 28–38] and 507 patients (55.6%) were men. Majority of patients were at an advanced stage of HIV disease: 261 (28.6%) with WHO stage 3 and 531 (58.2%) with WHO stage 4. The median baseline CD4 cell count was 49 cells/μL [IQR 15–148] (n=775). Women had a significant higher CD4 cell count than men (median 65 vs. 41 cells/μL, *P*=0.002). The median BMI (18.5 kg/m^2^ [IQR 16.8-20.6], n=904) and the median haemoglobin level (11.4 g/dL [IQR 9.7-13.1], n=530) did not strongly differ by gender (*P* = 0.07). The two main initial regimens were: stavudine/lamivudine/neviparine (Triomune®) (65.1%) and stavudine/lamivudine/efavirenz (33.1%).

**Table 1:** Patients’ characteristics at HAART initiation, Savannakhet, Lao PDR, 2003–2009

**Characteristic**^**a**^	**Men (N=507)**	**Women (N=405)**	***P***	**Overall (N=913)**
**Age (years)**				
Median [IQR]	33 [29 – 38]	32 [27 – 37]	0.003	32 [28 – 38]
**WHO clinical stage**				
Stage 4	316 (62.3%)	215 (53.1%)	< 0.001	531 (58.2%)
Stage 3	143 (28.2%)	117 (28.9%)		261 (28.6%)
Stage 2	35 (6.9%)	44 (10.9%)		79 (8.6%)
Stage 1	13 (2.6%)	29 (7.1%)		42 (4.6%)
**CD4 cell count (cells/μL)**^**b**^				
Median [IQR]	41 [12 – 130]	65 [20 – 166]	0.002	49 [15 – 148]
< 50	236 (55.3%)	155 (44.7%)		391 (50.5%)
50-199	144 (33.7%)	133 (38.3%)		278 (35.9%)
≥ 200	47 (11.0%)	59 (17.0%)		106 (13.6%)
**Body mass index (kg/m**^**2**^**)**^**c**^				
Median [IQR]	18.7 [17.1 – 20.7]	18.2 [16.4 – 20.5]	0.07	18.5 [16.8 – 20.6]
**Haemoglobin level (g/dL)**^**d**^				
Median [IQR]	11.6 [9.9 – 13.2]	11.3 [9.6 – 12.7]	0.07	11.4 [9.7 – 13.1]

^a^ 1 missing value on gender.

^b^ 138 missing values: 80 for men, 58 for women.

^c^ 9 missing values: 7 for men, 2 for women.

^d^ 383 missing values: 211 for men, 172 for women.

During HAART, the median CD4 cell gain increased strongly in the first year and attained +156 cells/μL [IQR 104–231] at 12 months (n=335). Then, it increased slowly from +191 cells/μL [IQR 121–269] at 18 months (n=336) to +294 cells/μL [IQR 162.5-405] at 42 months (n=152) before stabilizing.

The results of the random-intercept linear mixed model are reported in Table 2. Patients with a higher baseline CD4 cell count achieved a higher long-term CD4 reconstitution, indeed patients with CD4 cell count between 50 and 199 cells/μL (respectively more than 200 cells/μL) had on average +82 cells/μL (respectively +194 cells/μL) more than patients with less than 50 cells/μL at initiation after 2.5 years on HAART. However, the CD4 cells increased faster for patients with a lower baseline CD4 cell count (significant interaction between baseline CD4 cell count and time). Indeed, patients who had a baseline CD4 cell count < 50 cells/μL gained, on average per year, about +12 cells/μL and +20 cells/μL more than patients who had a baseline CD4 cell count between 50 and 199 cells/μL and patients who had more than 200 cells/μL, respectively. In addition, after adjusting for baseline CD4 count, women had on average +38 cells/μL more than men after 2.5 years on HAART. Furthermore, the significant effect of the interaction term between gender and time indicated that women gained on average about +15 cells/μL per year more than men. Finally, variance of the random-intercept represented about 35% of the total variance (p<0.001), showing an important heterogeneity between patients.

**Table 2:** Predictors of CD4 reconstitution identified by the random-intercept linear mixed model (N=774), Savannakhet, Lao PDR, 2003–2009

**Predictors**	**Coefficient**	**95% CI**
**Time since HAART initiation (year)**^**a**^		
Linear trend	25.15	(18.36; 31.93)*
Quadratic trend	−15.36	(−18.01; −12.71)*
Cubic trend	6.48	(5.04; 7.92)*
**Gender**^**b**^		
Intercept	38.54	(23.70; 53.38)*
Slope deviation	14.82	(9.52; 20.12)*
**CD4 at HAART initiation**^**c**^		
**Intercept**		
50–199 cells/μL	82.35	(66.74; 97.95)*
≥ 200 cells/μL	194.42	(169.29; 219.55)*
**Slope deviation**		
50–199 cells/μL	−11.64	(−17.12; −6.15)*
≥ 200 cells/μL	−20.10	(−29.45; −10.74)*

* *P* < 0.001.

^a^ Time was centered at 2.5 years on HAART.

^b^ Reference group: men.

^c^ Reference group: CD4 at HAART initiation < 50 cells/μL.

**Figure 1:**
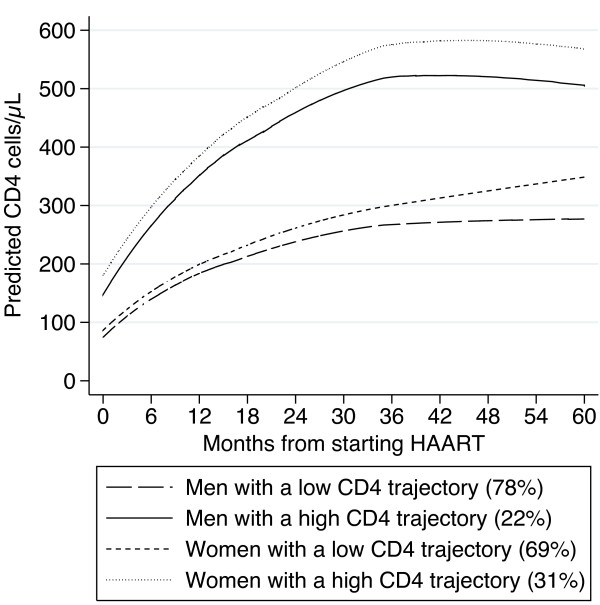
Latent CD4 trajectories for men and women on HAART, Savannakhet, Lao PDR, 2003–2009.

The latent trajectory analysis reveals both for men and women two typical latent trajectories of CD4 cells over time (Figure 1). The first latent trajectory represented 78% of men and 69% of women, with a low CD4 trajectory, achieving about 300 cells/μL after 60 months. The second latent trajectory represented 22% of men and 31% of women who experienced a high CD4 trajectory, reaching about 500 and 600 cells/μL after 60 months for men and women, respectively. The average membership probability was about 0.8 for patients with a low number of CD4 measurements, and tended to 1 for patients with higher number of CD4 measurements.

Analysis of appointments revealed that 85.6% of visits took place without delay and that the median delay was 2 days [IQR 1–7]. A significant interaction between gender and time (in years) indicated a better punctuality of appointments for women compared to men in the long-term on HAART (Odds Ratio of interaction between gender and time: 1.09; 95% CI 1.01-1.19).

**Figure 2:**
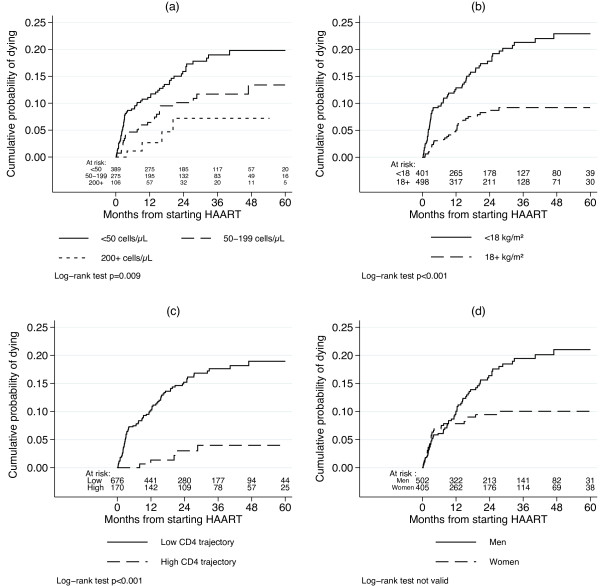
**Kaplan-Meier estimates of mortality on HAART, Savannakhet, Lao PDR, 2003–2009.** Estimates stratified by: CD4 at HAART initiation (**a**); BMI at HAART initiation (**b**); the latent CD4 trajectories (**c**); and gender (**d**).

The median participation time was 21.7 months [IQR 8.1-42.5] and the average interval between two visits was 1 month and 11 days. On HAART, 40 patients were lost to follow-up (4.4%), 98 were transferred to Vientiane (10.7%) and 107 died (11.7%). Cumulative probability of being lost to follow-up on HAART was 2.42% (95% CI 1.16-3.19%) at 12 months and 5.33% (95% CI 3.72-7.63%) at 30 months. Kaplan-Meier estimates of mortality attained 6.2% (95% CI 4.8-8.0%), 7.7% (95% CI 6.1-9.7%) and 9.1% (95% CI 7.3-11.3%) at 4, 9 and 12 months, respectively. Mortality differed by the following factors: CD4 cell count (Figure 2a, log-rank test: *P*=0.007) and BMI (Figure 2b, log-rank test: *P*<0.001) at baseline, and by the typical latent CD4 trajectories (Figure 2c, log-rank test: *P*<0.001), showing a higher mortality among patients with a low CD4 trajectory. In addition, mortality seems higher for men after 9 months on HAART (Figure 2d), but due to the violation of the PH assumption, the log-rank test was not valid and we can not conclude of a significant difference. Following our analysis plan, baseline CD4 cell count was not included in the multivariate Cox model, which showed that a high CD4 trajectory over time (Hazard Ratio 0.19, 95% CI 0.08-0.47), female gender after 9 months on HAART (HR 0.17, 95% CI 0.07-0.44) and a baseline BMI ≥ 18 kg/m^2^ (HR 0.39, 95% CI 0.25-0.60) were independently associated with a lower death rate (Table 3). However, gender before 9 months on HAART and attendance to clinical appointments were not associated with mortality.

**Table 3:** Adjusted mortality rate ratios (95% confidence interval) from Cox proportional-hazards model (N=840), Savannakhet, Lao PDR, 2003–2009

**Predictors**	**HR**	**95% CI**	***P***
**Latent CD4 trajectories**			
Low CD4 trajectory	1		
High CD4 trajectory	0.19	(0.08; 0.47)	< 0.001
**Gender before 9 months on HAART**			
Men	1		
Women	1.19	(0.69; 2.05)	0.53
**Gender after 9 months on HAART**			
Men	1		
Women	0.17	(0.07; 0.44)	< 0.001
**BMI at HAART initiation**			
< 18 kg/m^2^	1		
≥ 18 kg/m^2^	0.39	(0.25; 0.60)	< 0.001

Schoenfeld residuals.

Global chi-squared test: p=0.53; detailed chi-squared test: latent CD4 trajectories: p=0.09; gender before 9 months on HAART: p=0.64; gender after 9 months on HAART: p=0.89; BMI at HAART initiation: p=0.67.

## Discussion

Throughout this retrospective study, a better long-term immunologic reconstitution for women after HAART initiation was pointed out. Two typical latent trajectories of the CD4 cells for men and women were identified, showing that about 31% of women had a high CD4 trajectory against about 22% of men. In addition, a better survival was independently associated to female gender after 9 months and to patients with a high CD4 trajectory on HAART.

### Programme evaluation

A recent bulletin of the World Health Organization (WHO) states that, on average, 21% of the patients are lost to follow-up in the first 6 months after HAART initiation in resource-limited settings [23]. In Sub-Saharan Africa, a systematic review of HAART programmes shows that around 40% of patients were lost to follow-up after 2 years on HAART [24]. In addition, a recent meta-analysis of the mortality of patients LTFU in resource-limited settings reveals that 20 to 60% of the patients LTFU have died [25]. The present analysis indicates a low lost to follow-up rate showing a good compliance to the programme. Moreover, the high frequency of the visits at the hospital (on average every 1 month and 11 days) underlines the good monitoring of the cohort.

### Immune reconstitution

As it has been reported in other studies conducted in Southeast Asia, patients who start HAART were severely immunocompromised [15,16,18]. Without viral load measurements, WHO recommends to use CD4 reconstitution to assess the efficacy of treatment [26]. In this study, the median CD4 cells gain increases with time on HAART and this progression is comparable to the one observed in other low-income countries [15,18-20,27,28].

As in other studies, the baseline CD4 cell count is a strong predictor of immune reconstitution, indicating that patients with higher baseline CD4 cell count achieve a better immune recovery [29,30]. In addition, our findings show that, overall, the CD4 cells increase faster for the most immunocompromised patients. This suggests that, in the long-term, the most immunocompromised patients, surviving the early phase on HAART, could restore their immunity as well as the other patients. Taking together, these results argue in favour of an earlier access to HAART and of an improvement in diagnosis and treatment of opportunistic infections during first months on treatment.

Several studies on large cohorts and long follow-up have failed to associate gender and immune reconstitution [31,32]. This study shows that women achieve a better immune recovery on HAART independently of the baseline CD4 cell count. This could be explained by better adherence for women, but we are unable to check this hypothesis because of the lack of adherence measurements. Although gender is generally not associated with adherence, comprehensive studies in Burkina Faso have shown a better use of health care facilities for women in the West of Africa as well as a better communication with health care workers on disease and treatment issues and argue in favour of better adherence behaviour among women [33-35]. Moreover, a higher long-term adherence to HAART among women has recently been shown in Senegal [36]. In this study, we have shown that women attend more regularly follow-up visits over time than men. This indicator, which could be used as a proxy of adherence, argues in favour of a better adherence for women. Another explanation could be that men who are frequently migrant worker do not adhere entirely to HAART because they could not refill on time [10-12]. In addition, a recent study in Thailand shows that women have more and earlier access to antiretroviral treatments [37]. Other factors like the stigma of HIV infection, family responsibilities, work or homophobia could impact on treatment access for men [38-40]. It has been shown that HIV-negative women had higher CD4 cell count than men in Africa [41]. However, it does not impact on HAART eligibility. Although our model is adjusted for baseline CD4 cell count, a physiological difference in CD4 counts could explain a steeper slope for women to return to their immunological equilibrium.

The latent trajectory analysis has identified two patterns of the CD4 evolution both for men and women. Overall, about a quarter of the patients followed a similar high CD4 trajectory, and about three quarters a lower one. About a third of women achieved a high CD4 trajectory whereas about a fifth of men achieved it. These results are in line with the mixed model.

The prevalence of the two latent trajectories reflects the distribution of baseline CD4 cell count. Indeed, patients with a low CD4 cell count at HAART initiation are more likely to follow a low CD4 trajectory. This explains why the prevalence of the first latent trajectory is high. Strength of this approach is that it describes the typical evolution of the CD4 cells in that cohort and it captures the effect of the low baseline CD4 count. The high membership probability observed even for patients with a low number of CD4 measurements allows us to assign patients to a unique latent trajectory with confidence. This is a new approach to explore different patterns of the CD4 evolution and it could be generalized to other longitudinal studies to reveal different patients’ behaviour over time.

### Mortality

Half of the deaths occur in the first four months after HAART initiation, showing a higher early mortality than the one generally observed in low-income countries. A recent scale-up of national antiretroviral therapy programmes in the Asia Pacific region shows that 50% of mortality occurs in the first 6 months of therapy [42]. High early mortality has also been reported in sub-Saharan Africa [43-46]. However, after 1 year on treatment, mortality is about 9% which is similar to other low-income countries [15,19,28,47,48]. A pooled analysis from 18 developing countries has assessed the risk of dying after 1 year at 5.8%, which is about twice as low as in this cohort, as in other settings [49,50]. This early mortality could be explained by the advanced stage of infection and the low CD4 cell count of patients at enrollment, due to late presentation of patients [46]. As it has already been reported, poor adherence could be associated with increase in mortality [48,51,52].

Mortality differs by patients’ baseline characteristics such as CD4 cell count and BMI. These relationships have already been largely described both in low-income and high-income countries [15,18,28,44,45,47,53-55].

As shown in an observational cohort in South Africa, mortality is strongly associated with immune reconstitution during HAART [56]. Indeed, mortality differs by the two latent CD4 trajectories with a fivefold risk of death for the three quarters of patients following a low CD4 trajectory. Introducing these trajectories as a covariate of the Cox model provide a new way to explore the link between mortality and CD4 evolution on HAART, as it captures simultaneously the baseline and the reconstitution itself.

Our study also reveals a higher mortality among men. This result is also reported in other studies [57-63]. Men are more immunocompromised at HAART initiation which suggests a late presentation, as reported in South Africa [64]. Lower adherence and higher mobility among men are also mentioned by programme managers in Cambodia [58]. Here, the difference in mortality between men and women appears only after 9 months on HAART. It is important to highlight that this effect is independent of the latent CD4 trajectories and BMI. This result could point out sub-optimal care and treatment of clinical events occurring among men after the first year on treatment.

### Limitations of the study

This study presents some limitations. First, we have no information about outcomes of patients lost to follow-up and transferred to Vientiane, which could bias the estimated immune recovery and mortality in the cohort. Adjusting mortality for loss to follow-up could correct the estimates, but due to the low lost to follow-up rate in this cohort, we did not use this method [65]. Then, because of technology availability and costs, viral load measurements were not available at that time. In addition, no data on adherence to antiretroviral was available.

## Conclusions

This MSF programme where 913 patients initiated HAART with a very severe immuno-suppression reveals an important heterogeneity between patients and better outcomes for women. Indeed, a quarter of the patients present a high CD4 trajectory and women experience a better immune recovery and lower mortality than men. This study provides additional evidence for differences between men and women, once on HAART and, in particular, a difficulty for men to maintain a long-term compliance to treatment. Moreover, this study recommends that clinicians and healthcare providers have to pay special attention to men during treatment.

## Competing interests

The authors declare that they have no competing interests.

## **Authors**’ **contributions**

MB designed the analyses, analyzed the data and interpreted the results, wrote the paper and submitted the final version. KS designed the study and collected the data. PP designed the study, interpreted the results and reviewed the manuscript. AHS designed the study and collected the data. LC designed the study, interpreted the results and reviewed the manuscript. AC designed the study, collected the data, interpreted the results and reviewed the manuscript. CP designed the study, interpreted the results and reviewed the manuscript. RE designed the analyses, interpreted the results and reviewed the manuscript. JFE coordinated the research, designed the analyses, interpreted the results and reviewed the manuscript. All authors read and approved the final manuscript.

## Pre-publication history

The pre-publication history for this paper can be accessed here:

http://www.biomedcentral.com/1471-2334/13/27/prepub
